# Cerebral Venous Sinus Thrombosis Presenting as Focal Seizures: A Case Report and Review of Neuroimaging Findings

**DOI:** 10.7759/cureus.77246

**Published:** 2025-01-10

**Authors:** Hasham Ramzan, Aakash Mahajan

**Affiliations:** 1 Medical Imaging, North Canberra Hospital, Canberra, AUS; 2 Radiology, Qscan Radiology Clinics, Canberra, AUS

**Keywords:** brain mri flair signals, cerebral venous sinus thrombosis (cvst), ct venogram, non-contrast ct, seizure

## Abstract

Cerebral venous sinus thrombosis (CVST) is a rare but potentially life-threatening condition that can present with a wide range of neurological symptoms. This case report reviews a 56-year-old male patient who initially presented with focal seizures, highlighting the importance of considering CVST in the differential diagnosis of new-onset seizures, even in the absence of typical risk factors. The case also emphasizes the challenges in interpreting neuroimaging findings and the value of maintaining a high index of suspicion for CVST.

## Introduction

Cerebral venous sinus thrombosis (CVST) is a rare cerebrovascular disorder, accounting for approximately 0.5%-1% of all strokes [1]. The clinical presentation of CVST can vary considerably, from headaches to focal neurological deficits and seizures. Seizures occur in approximately 40% of CVST cases and can be the presenting symptom in up to 12%-31.9% of patients [2,3]. This case report presents a 56-year-old male patient with no significant medical history who developed focal seizures as the initial manifestation of CVST. The case emphasizes the importance of maintaining a high index of suspicion for CVST in patients presenting with new-onset seizures, particularly when initial neuroimaging appears unremarkable.

## Case presentation

A 56-year-old male patient presented to the emergency department with a first-time generalized tonic-clonic seizure. The patient reported a one-week history of left-sided headache and altered sensation in his right forearm prior to the seizure. He also described visual disturbances, including circles in his vision and possible blind spots, for the past six to 12 months.

The patient had a history of controlled hypercholesterolemia managed with rosuvastatin and exercise-induced asthma treated with salbutamol as needed. He had no significant cardiovascular or metabolic diseases and was allergic to peanuts. The patient consumed six to seven standard alcoholic drinks per week and was a non-smoker. There was no significant family history of stroke.

On initial neurological examination, the patient demonstrated a slight right facial droop with normal extraocular movements and power in the upper and lower limbs. Reflexes were normal, with a possible slight loss of cerebellar function in the right arm.

**Figure 1:**
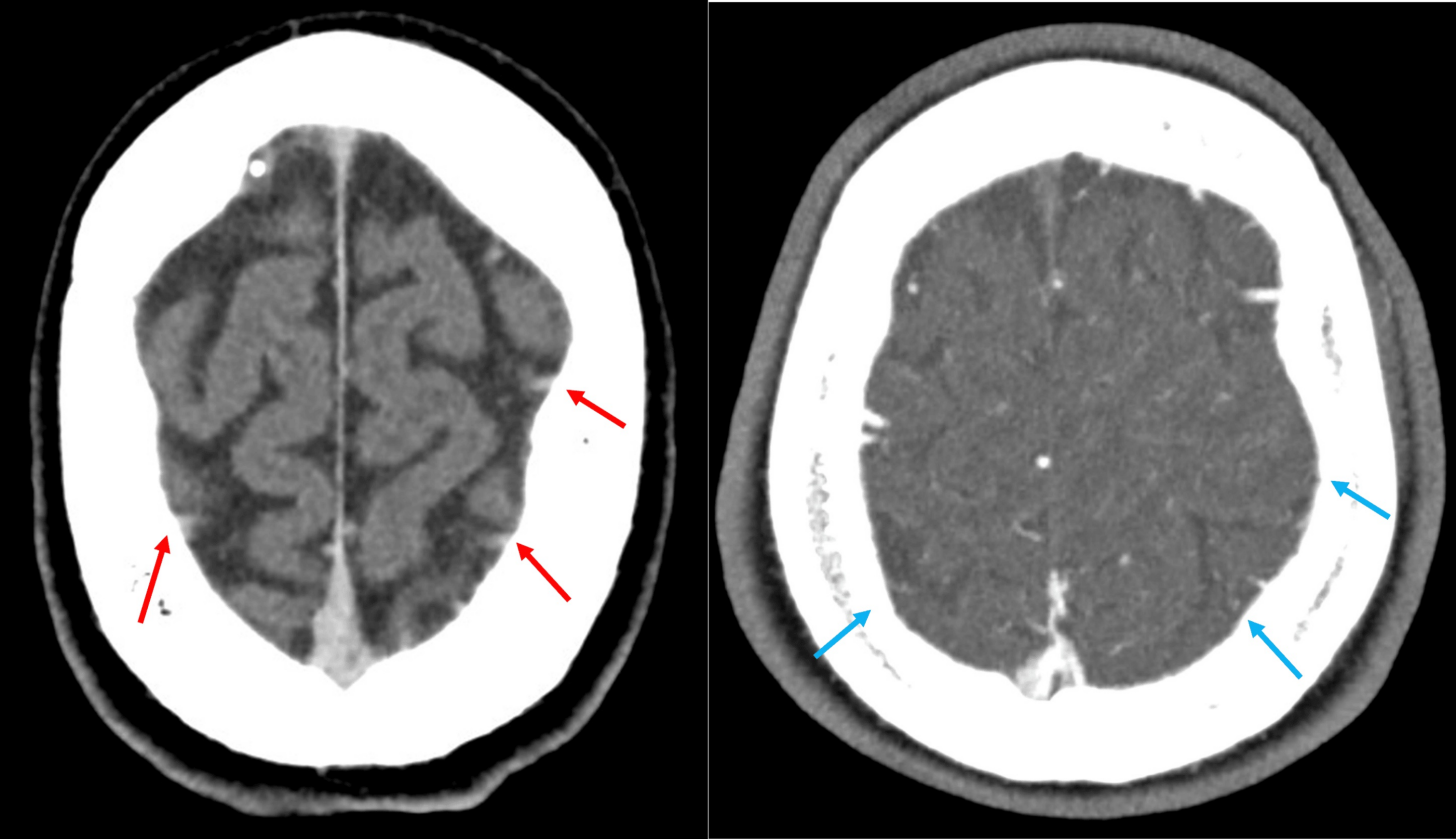
Initial CT brain (15/09/24) image reported showing no acute abnormalities. The red arrows indicate areas of hyperdensity that, in retrospect, could represent thrombi on a non-contrast scan. The blue arrows indicate areas where there is no flow on the CT angiogram, demonstrated by a lack of hyperdense white contrast, further indicating the presence of a thrombus. Although not a dedicated venogram, there is a filling of venous sinuses visible superior to the blue arrows.

The initial investigations for this case were comprehensive but yielded unremarkable results. A computed tomography (CT) angiogram of the brain and neck was performed, revealing no acute abnormalities, as illustrated in Figure 1. The electrocardiogram (ECG) showed a normal sinus rhythm, while the chest X-ray appeared normal. Blood tests were also conducted and found to be within normal limits. These initial findings, while reassuring, did not provide a clear explanation for the patient's presenting symptoms, highlighting the importance of further investigation and follow-up in cases where clinical suspicion remains high despite seemingly normal initial results.

**Figure 2:**
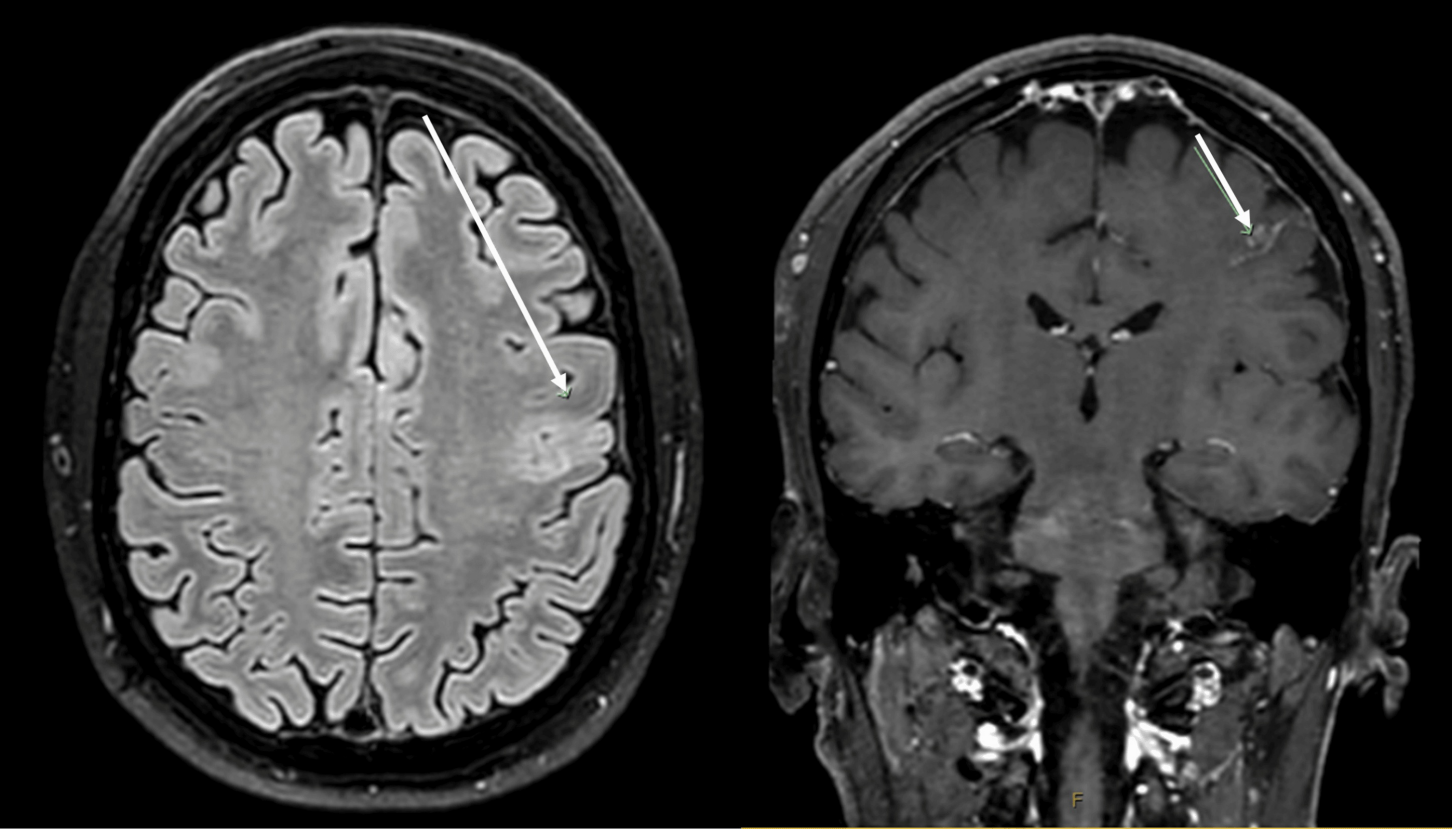
Initial MRI of the brain (16/09/24) showed the focal area of altered F2/fluid-attenuated inversion recovery (FLAIR) signal intensity on the left. The image on the right is a post-contrast T1-weighted (T1W) sequence.

**Figure 3:**
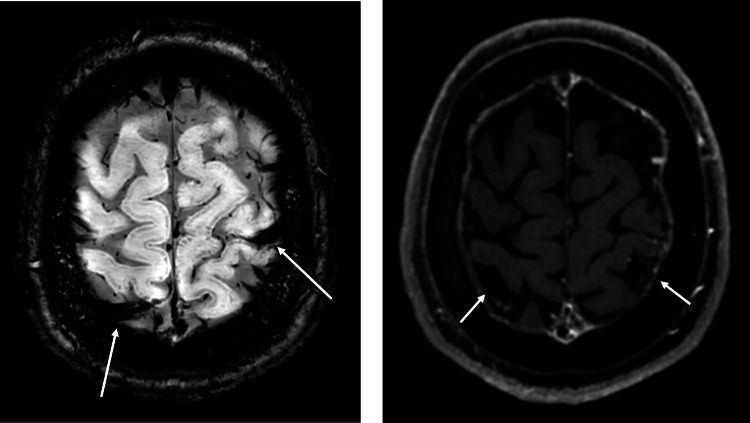
Magnetic resonance susceptibility-weighted imaging (MR SWIP) (16/09/24) on the left showed abnormally thickened blood vessels suspicious for thrombi. Magnetic resonance gadolinium (MR GAD) contrast on the right side showed areas of reduced contrast enhancement, corresponding to areas on MR SWIP. This was not commented on in the initial report.

The patient was admitted to the neurology ward for further evaluation. Magnetic resonance imaging (MRI) of the brain with contrast was performed on 16/09/2024 (Figure 2 and Figure 3), revealing a focal area of altered signal intensity in the left inferior frontoparietal lobe around the inferior portion of the central sulcus, measuring approximately 22 mm in maximum dimension.

The differential diagnosis at this point included an infiltrating low-grade neoplasm, parenchymal changes related to an adjoining small vascular malformation, or postictal changes. The patient was started on levetiracetam (Keppra) 1 g twice daily for seizure management. A lumbar puncture was performed, of which the cerebrospinal fluid (CSF) analysis was unremarkable (glucose 3.8 mmol/L (2.5-4.4 mmol/L), protein 336 mg/L (150-450 mg/L)).

**Figure 4:**
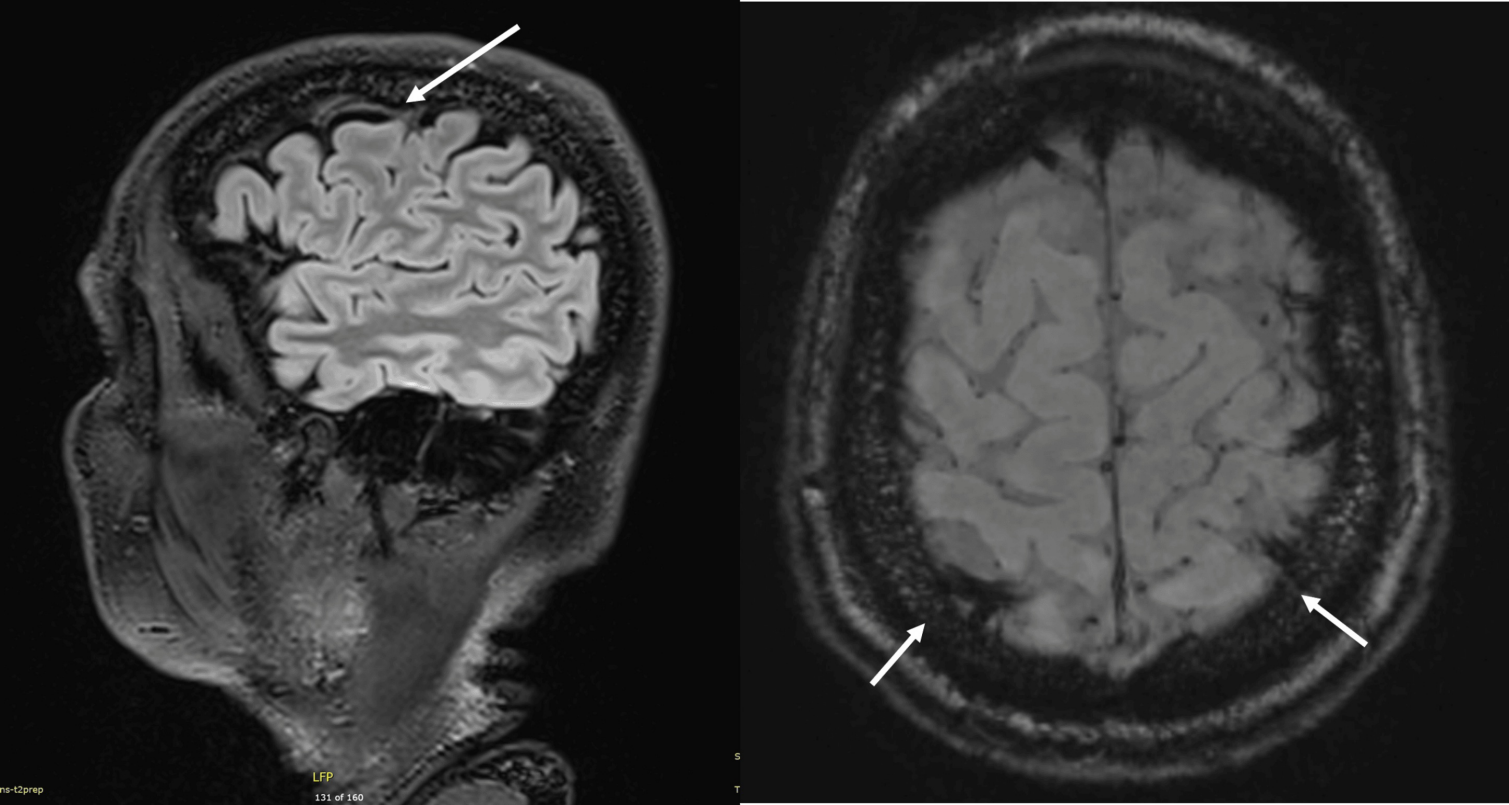
A follow-up MRI of the brain (19/09/24) image showed a reduced T2/fluid-attenuated inversion recovery (FLAIR) signal indicated on the image on the left. Magnetic resonance susceptibility-weighted imaging again showed abnormally vessel thickening corresponding to thrombi.

During the hospital stay, the patient experienced intermittent right facial twitching and altered sensation, managed with as-needed doses of levetiracetam. A repeat MRI brain was performed on 19/09/2024 (Figure 4) following neurosurgery advice; this showed a reduced T2/fluid-attenuated inversion recovery (FLAIR) signal around the left inferior central sulcus, with no significant elevated blood flow. These findings were more consistent with postictal changes rather than a lesion.

The patient was discharged on 17/09/2024 with a plan for outpatient follow-up with neurology and neurosurgery.

**Figure 5:**
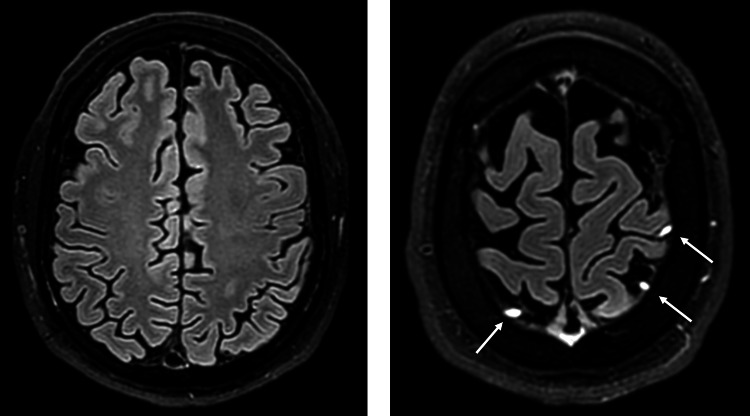
The third brain MRI (01/11/24) image showed the resolution of T2/fluid-attenuated inversion recovery (FLAIR) hyperintensity in the image on the left. The right-sided image also showed T2/FLAIR hyperintensity in regions previously corresponding to areas of thrombus.

During follow-up visits, the patient reported occasional right facial twitching and paraesthesia. Clobazam was added to his treatment regimen for better control of focal seizure activity. A third MRI brain was performed on 31/10/2024 (Figure 5), which showed resolution of the previously noted T2/FLAIR hyperintensity but revealed a new hyperintensity in T2/FLAIR imaging in the regions corresponding to areas of thrombus. This finding, along with the clinical presentation, raised suspicion for CVST.

**Figure 6:**
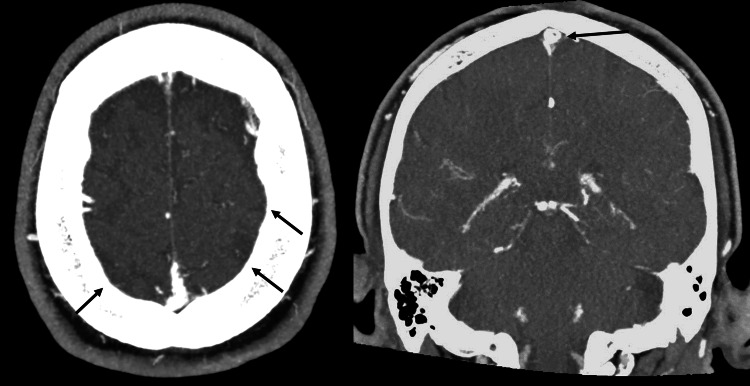
A CT venogram image showed bilateral cerebral venous sinus thrombosis. Note the image on the left is similar in appearance to the computed tomography angiogram (CTA) performed on 15/9/24 in Figure 1. A subtle filling defect was visible in the right lateral superior sagittal sinus, suggestive of a non-occlusive thrombus indicated by the arrow on the right panel.

A CT venogram was subsequently performed on 05/11/2024 (Figure 6), confirming the presence of bilateral CVST. The patient was started on rivaroxaban 15 mg twice daily, which was well tolerated. His antiepileptic medications were continued, with a plan to taper if seizure-free.

## Discussion

This case highlights the importance of considering CVST in the differential diagnosis of new-onset seizures, even in patients without typical risk factors. The initial presentation with focal seizures and the evolving neuroimaging findings demonstrate the challenges in diagnosing CVST.

In retrospect, signs of CVST are evident on previous CT and MRI imaging; however, this case highlights the difficulty in interpreting MRI and CT scans, with multiple radiologist reports required before a diagnosis is reached. As the thrombus evolves over time, so do the findings on imaging, and in retrospect, it’s often obvious to see findings; however, at the moment, it can be easy to overlook.

The initial area of hyperintensity on T2/FLAIR MRI (as seen in Figure 2) likely corresponds to an area of infarct, which then resolves with the subsequent imaging. As the thrombus matures over time, it becomes more evident, as seen on the T2/FLAIR MRI in Figure 5.

This case underscores several important considerations in the diagnosis and management of CVST. Firstly, it demonstrates that CVST can manifest with a diverse array of symptoms, including isolated seizures, which may not immediately suggest vascular pathology. This variability in presentation emphasizes the need for clinicians to maintain a high index of suspicion for CVST, even in the absence of typical signs and symptoms. Secondly, the case illustrates that initial neuroimaging, including CT and MRI, may provide subtle indications of CVST that require careful interpretation and appropriate follow-up imaging. It is crucial to recognize that the resolution of initial MRI abnormalities does not necessarily rule out underlying pathology and may, paradoxically, serve as a clue to the diagnosis of CVST. This highlights the dynamic nature of neuroimaging findings in CVST and the importance of serial imaging in some cases. Lastly, this case emphasizes the value of considering CT venography or MR venography in cases of unexplained seizures or evolving neurological symptoms, even when initial imaging appears unremarkable. This approach can lead to earlier diagnosis and treatment of CVST, potentially improving patient outcomes.

## Conclusions

This case report underscores the importance of maintaining a broad differential diagnosis in patients presenting with new-onset seizures. It demonstrates that CVST can present with isolated seizures and highlights the potential for evolving neuroimaging findings. Clinicians should have a low threshold for dedicated venous imaging in cases of unexplained seizures or evolving neurological symptoms, even when initial brain imaging appears normal or non-specific.
